# Ferritin modifies the relationship between inflammation and arterial stiffness in hypertensive patients with different glucose tolerance

**DOI:** 10.1186/s12933-020-01102-8

**Published:** 2020-08-05

**Authors:** Angela Sciacqua, Ettore Ventura, Giovanni Tripepi, Velia Cassano, Graziella D’Arrigo, Stefanos Roumeliotis, Raffale Maio, Sofia Miceli, Maria Perticone, Francesco Andreozzi, Giorgio Sesti, Francesco Perticone

**Affiliations:** 1 Department of Medical and Surgical Sciences, University Magna Græcia of Catanzaro, Campus Universitario di Germaneto, V.le Europa, 88100 Catanzaro, Italy; 2 Institute of Clinical Physiology (IFC-CNR), Clinical Epidemiology and Physiopathology of Renal Diseases and Hypertension of Reggio Calabria, Reggio Calabria, Italy; 3 Division of Nephrology and Hypertension, 1st Department of Internal Medicine, AHEPA Hospital, School of Medicine, Aristotle University of Thessaloniki, Thessaloniki, Greece; 4 Department of Experimental and Clinical Medicine, University Magna Græcia of Catanzaro, Catanzaro, Italy; 5 Department of Clinical and Molecular Medicine, University of Rome-Sapienza, Rome, Italy

**Keywords:** Ferritin, Iron, Arterial stiffness

## Abstract

**Background:**

Ferritin, a crucial element for iron homeostasis, is associated with chronic diseases characterized by subclinical inflammation such as essential arterial hypertension and type 2 diabetes mellitus (T2DM), showing a prognostic value in different clinical settings. We investigated whether ferritin is associated with arterial stiffness (AS), an early indicator of atherosclerosis, and if it could act as effect modifier on the relationship between inflammation and AS in hypertensive patients with different glucose tolerance.

**Methods:**

We enrolled 462 newly diagnosed untreated hypertensive (HT) patients. All subjects underwent an oral glucose tolerance test. Insulin sensitivity was assessed by MATSUDA index and ferritin levels were estimated by immunoradiometric assay. AS was defined by carotid-femoral pulse wave velocity (PWV).

**Results:**

Out of 462 patients, 271 showed normal glucose tolerance (HT/NGT), 146 impaired glucose tolerance (HT/IGT) and 45 were diabetic (HT/T2DM). Iron levels significantly decreased and transferrin and ferritin significantly increased from the first to the third group. PWV values were significantly higher in HT/IGT and HT/T2DM patients. PWV was related directly with ferritin, high sensitivity C reactive protein (hs-CRP), transferrin, and inversely with MATSUDA index. Ferritin resulted the strongest determinant of PWV explaining a 14.9% of its variation; moreover it was a strong modifier of the relationship between hs-CRP and PWV. The estimated augmentation in PWV portended by a fixed increase in hs-CRP, was higher across increasing values of ferritin.

**Conclusion:**

Ferritin represents an independent risk factor of arterial stiffness in our study population and a strong effect modifier on the relationship between inflammation and PWV. However, further studies are needed to fully elucidate the potential role of this biomarker in human atherosclerosis.

## Background

Iron plays a pivotal role in preservation of the biological systems but it is also true that an excess of this cation may lead to reactive oxygen species (ROS) production thus promoting cellular and tissue damage [1, 2].

Ferritin is an ubiquitous protein which represents not only a crucial element of iron homeostasis regulation but also the most used biomarker of iron deficiency [3]. Moreover, serum ferritin is a well-known acute phase protein reflecting the degree of acute and chronic inflammation and compelling evidence suggest a potential active role of ferritin in chronic inflammatory diseases [4–6]. Accordingly, several studies report a direct association between serum ferritin levels and chronic inflammation of mild degree [7–11]. In particular, serum ferritin levels resulted to be directly related to insulin-resistance and incident risk of type 2 diabetes mellitus (T2DM), independently of traditional risk factors [7, 8], especially when high-sensitivity C reactive protein (hs-CRP) is increased [9]. Furthermore, in various populations it has been demonstrated that ferritin is associated with a high risk of arterial hypertension development [10, 11], this latter being the most common risk factor of cardiovascular (CV) events and disability as well [12].

It is well known that arterial stiffness is increased in patients with T2DM and arterial hypertension [13, 14] and represents not only an early biomarker of atherosclerotic process but also an independent risk factor of CV outcomes in these conditions [15, 16]. In particular, increased carotid-femoral pulse wave velocity (PWV), the gold standard parameter of arterial stiffness evaluation, reflects the disparity between peripheral and aortic blood pressure (BP). A recent meta-analysis shows 8 hat 1 m/s increase in PWV is associated with a 15% risk increase of CV mortality in about 16,000 individuals followed up for a mean of 8 years [17]. Although there is some evidence of an independent association between hyperferritinemia and arterial stiffness in disparate populations [18–21], data in hypertensive patients are few.

In a large group of naïve hypertensive patients with different glucose tolerance status, we addressed the following hypotheses: (1) to assess the relationships between ferritin levels in the normal range with PWV and glucose tolerance and to evaluate whether such associations are paralleled by a concomitant increase in hs-CRP; (2) to test whether circulating levels of ferritin are related to PWV independently of insulin sensitivity MATSUDA index and other potential confounders; (3) to verify whether inflammation (as measured by hs-CRP) acts as an effect modifier of the link between ferritin and PWV.

## Methods

The Ethical Committee approved the protocol and informed written consent was obtained from all participants. All the investigations were performed in accordance with the principles of the Declaration of Helsinki.

### Study population

The study group consisted of 462 Caucasian newly diagnosed hypertensive patients, 216 men and 246 women aged 49.6 ± 12.2 years, participating in the Catanzaro Metabolic Risk factors Study. All subjects underwent physical examination and review of their medical history. Causes of secondary hypertension were excluded by appropriate clinical and biochemical tests. Other exclusion criteria were history or clinical evidence of coronary and valvular heart disease, congestive heart failure, peripheral vascular disease, chronic inflammatory diseases, anemia, gastrointestinal diseases with malabsorption, history of any malignant disease, history of alcohol or drug abuse, liver or kidney failure and diabetes already diagnosed. No patient had ever been treated with antihypertensive drugs. All subjects underwent anthropometrical evaluation with measurements of weight, height, and body mass index (BMI). After 12-h fasting, a 75 g oral glucose tolerance test (OGTT) was performed with 0, 30, 60, 90 and 120 min sampling for plasma glucose and insulin. Glucose tolerance status was defined on the basis of OGTT using the World Health Organization (WHO) criteria.

### Blood pressure measurements

Measurements of clinic BP were obtained in the left arm of the patients, in supine position, after 5 min of quiet rest, with a aneroid sphygmomanometer. A mean value of at least three BP readings were taken in three different visits at least 2 weeks apart. Systolic and diastolic BP were recorded at the first appearance (phase I) and the disappearance (phase V) of Korotkoff sounds, respectively. A value of clinic systolic BP (SBP) ≥ 140 mmHg and/or diastolic BP (DBP) ≥ 90 mmHg identified patients as hypertensive according to current guidelines [22]. In addition, pulse pressure (PP) was defined as the difference between SBP and DBP.

### Laboratory determinations

All blood samples were obtained after overnight fasting. Serum ferritin levels (10–290 ng/ml) were measured using an immunoturbidimetric assay (Roche Diagnostics, Indianapolis, IN, USA). The serum iron (50–150 µg/dl) was measured with a colorimetric method and transferrin (2–4 g/l) by a nephelometric assay, while the percentage of saturated transferrin iron binding capacity (TIBC) was measured using a colorimetric test (Roche Diagnostics, Cobas 8000, Switzerland). Hemoglobin was determined using an automated particle counter (Siemens Healthcare Diagnostics ADVIA^®^ 120/2120 Haematology System, Milan, Italy).

Plasma glucose was measured by the glucose oxidation method (Beckman Glucose Analyzer II; Beckman Instruments, Milan, Italy). Triglyceride, total, low- (LDL) and high-density lipoprotein (HDL) cholesterol concentrations were evaluated by enzymatic methods (Roche, Basel, Switzerland). Serum insulin levels were determined by a chemiluminescence-based assay ((Immulite ^®^, Siemens, Italy).). Insulin sensitivity was evaluated using the Matsuda index [insulin sensitivity index (ISI)], calculated as follows: 10,000/square root of [fasting glucose (millimoles per liter) × fasting insulin (milliunits per liter)] * [mean glucose * mean insulin during OGTT]. The Matsuda index is strongly related to euglycemic hyperinsulinemic clamp that represents the gold standard test for measuring insulin sensitivity [23].

Alanine aminotransferase (ALT) and aspartate aminotransferase (AST) levels were determined using the alpha-ketoglutarate reaction, and γ-glutamyltransferase (γGT) levels with the l-γ-glutamyl-3-carboxy-4-nitroanilide rate method (Roche, Basel, Switzerland). High sensitivity CRP (hsCRP) were measured by automated instrument (CardioPhase ^®^ hsCRP, Milan, Italy). Finally, creatinine levels were measured by Jaffe methodology and estimated glomerular filtration rate (e-GFR) was computed by using the chronic kidney disease epidemiology (CKD-EPI) collaboration equation [24].

### Arterial stiffness evaluation

All measurements were obtained using a validated system (Sphygmocor™; AtCor Medical, Sydney, Australia) that utilizes high-fidelity applanation tonometry (Millar) and an appropriate computer software for the evaluation of pressure wave (Sphygmocor™). At first, the pressure calibration was achieved by the non-invasive automatic recording of supine brachial artery BP in the dominant arm after a 30 min of rest (Dinamap Compact T; Johnson & Johnson Medical Ltd, Newport, UK). In particular, BP was measured five times over 10 min and the mean of the last three determinations was considered for calibration. On the radial artery of the dominant arm, the pressure wave was recorded as the average of single waves consecutively obtained for 8 s. Pressure wave determinations were considered reliable only if the variation of peak and bottom pressures of pressure waves was < 5%. The central pressure wave assessment was automatically derived from the radial measurements by a generalized transfer function [25]. Moreover, central waveforms were evaluated to identify the time to peak/shoulder of the first (T1) and second (T2) pressure wave elements during systole. The pressure at the peak/shoulder of T1 was classified as outgoing pressure wave height (P1), the pressure at the peak/shoulder of T2 was defined as the reflected pressure wave height (P2), either as an absolute value or as percent of ejection duration. Augmentation pressure (AP) was defined as difference between P2–P1, and augmentation index (AI) as [AP/pulse pressure (PP)] * 100. Aortic pulse wave velocity (PWV) was measured from carotid and femoral pressure waveforms. Carotid to femoral transit time (ΔT) was calculated from the foot-to-foot time delay between carotid and femoral waveforms. The distance between the landmarks of the sternal notch and femoral artery was considered to evaluate the path length between the carotid and femoral arteries (L), and PWV calculated as L/ΔT.

### Statistical analysis

Data are reported as mean and standard deviation or as absolute and percent frequency, as appropriate. Comparisons among more than two groups were performed by One Way ANOVA, followed by a Bonferroni post hoc between-groups comparison. Chi-squared test was utilized for categorical variables. The association between continuous variables was assessed by Pearson product moment correlation coefficient (r) and p value. The relationship between gender and PWV was investigated by point biserial correlation coefficient and p value. As potential confounders for the relationship between ferritin and PWV we considered all variables listed in Table 1 and those associated with this biomarker with p ≤ 0.05 at univariate analyses (see last column in Table 1) were considered to be introduced into the multiple linear regression model. To avoid collinearity, we did not include iron and transferrin into the multiple liner regression model because these two variables lied into the same causal pathway between ferritin and PWV. Similarly, we did not adjust for insulin to avoid collinearity between this variable and the MATSUDA index. To assess the variance of PWV explained by each covariate into the model, we calculated the squared of the partial correlation coefficient. The effect modification by ferritin on the relationship between hs-CRP and PWV was investigated by introducing into the same linear regression model ferritin (the effect modifier), hs-CRP and their interaction term (ferritin * hsCRP) as well as a series of potential confounders. The estimated increase of PWV associated to a fixed increase in CRP (+ 1 mg/l) across quartiles of ferritin (36 ng/ml, 86 ng/ml, and 159 ng/ml) was investigated by the standard linear combination method [26]. In multiple linear regression models, data were expressed as standardised regression coefficients (beta) and p value. All statistical analyses were performed using SPSS version 22 for Windows (Chicago, Illinois, USA) and STATA statistical package (version 13, Texas, USA).

**Table 1:** Characteristics of the study population according to glucose tolerance and linear correlational analysis with PWV

Variables	All (n = 462)	HT/NGT (n = 271)	HT/IGT (n = 146)	HT/T2DM (n = 45)	p	PWV versus (r, p)
Gender, m/f	216/246	107/164	76/70	33/12	0.098	0.088 (p = 0.058)
Age, years	49.6 ± 12.2	49.5 ± 13.7	49.7 ± 10.1	49.5 ± 8.4	0.978	0.247 (p < 0.001)
BMI, kg/m^2^	28.9 ± 4.8	28.4 ± 4.4	29.3 ± 5.5	30.5 ± 4.7	0.014	0.014 (p = 0.772)
Fasting glucose, mg/dl	96.0 ± 14.1	91.0 ± 10.1	98.3 ± 13.0	118.8 ± 14.4	< 0.0001	0.068 (p = 0.143)
Fasting insulin, µU/ml	13.3 ± 6.6	12.5 ± 5.4	14.1 ± 7.4	15.4 ± 9.6	0.004	0.324 (p < 0.001)
MATSUDA	64.1 ± 45.3	70.9 ± 47.3	55.9 ± 40.9	49.6 ± 38.2	< 0.0001	− 0.425 (p < 0.001)
LDL cholesterol., mg/dl	127.1 ± 41.1	126.4 ± 40.8	127.1 ± 36.1	131.1 ± 55.5	0.775	− 0.034 (p = 0.467)
HDL cholesterol, mg/dl	51.9 ± 14.6	53.9 ± 14.6	49.8 ± 14.7	46.6 ± 11.7	0.001	− 0.100 (p = 0.032)
Triglycerides, mg/dl	128.9 ± 75.6	121.4 ± 66.1	138.1 ± 89.9	143.8 ± 74.1	0.037	0.063 (p = 0.177)
Smokers, n (%)	95 (20.6)	56 (20.7)	30 (20.5)	9 (20)	0.850	− 0.013 (p = 0.777)
Hemoglobin, g/dl	13.8 ± 1.2	13.8 ± 1.1	13.9 ± 1.2	13.4 ± 1.2	0.028	0.091 (p = 0.05)
Serum iron, µg/dl	80.7 ± 28.8	85.8 ± 31.3	76.3 ± 22.9	64.2 ± 21.8	< 0.0001	− 0.258 (p < 0.001)
Ferritin, ng/ml	106.6 ± 81.4	92.2 ± 78.6	120.7 ± 78.7	147.8 ± 85.9	< 0.0001	0.499 (p < 0.001)
Transferrin, g/l	2.6 ± 0.8	2.5 ± 0.6	2.7 ± 0.9	2.9 ± 0.9	< 0.0001	0.108 (p = 0.02)
hs-CRP, mg/l	2.6 ± 1.7	2.3 ± 1.6	2.8 ± 1.7	3.4 ± 1.8	< 0.0001	0.334 (p < 0.001)
TIBC, µg/dl	330.9 ± 87.2	314.8 ± 60.6	347.8 ± 110.6	373.2 ± 111.6	< 0.0001	0.086 (p = 0.064)
AST, U/l	23.9 ± 10.8	22.2 ± 9.3	25.2 ± 11.7	30.5 ± 13.2	< 0.0001	0.112 (p = 0.016)
ALT, U/l	27.1 ± 12.9	25.4 ± 12.1	28.3 ± 12.4	32.8 ± 17.2	0.001	0.084 (p = 0.07)
γGT, U/l	29.9 ± 15.7	27.2 ± 14.7	32.7 ± 16.6	36.8 ± 14.1	< 0.0001	0.095 (p = 0.041)
e-GFR, ml/min/1.73 m^2^	99.9 ± 23.2	102.4 ± 23.9	97.9 ± 20.4	91.6 ± 25.3	0.007	− 0.246 (p < 0.001)

Data are mean and standard deviation, absolute and percent frequency

*H* hypertensive, *NGT* normal glucose tolerance, *T2DM* type 2 diabetes mellitus, *PWV* pulse wave velocity, *BMI* body mass index, *LDL* low density lipoproteins, *HDL* high density lipoproteins, *hs-CRP* high sensitivity C reactive protein, *TIBC* transferrin iron binding capacity, *AST* aspartate aminotransferase, *ALT* alanine aminotransferase, *γGT* γ-glutamyltransferase, *e-GFR* estimated glomerular filtration rate

## Results

### Study population

**Fig. 1:**
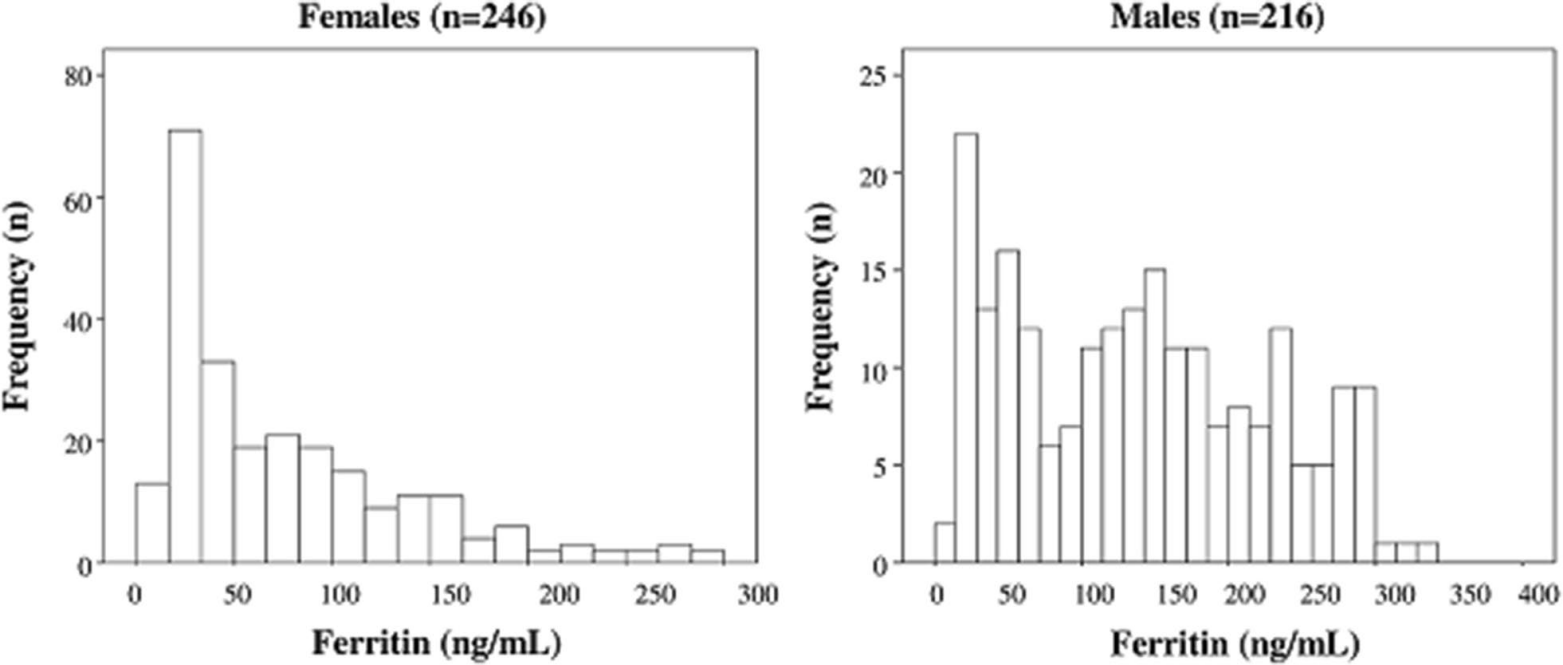
Distribution (histograms) of ferritin in males and females

Out of 462 hypertensive (HT) patients examined by OGTT, 271 (58.7%) showed normal glucose tolerance (HT/NGT), 146 (31.6%) impaired glucose tolerance (HT/IGT) and 45 (9.7%) type 2 diabetes mellitus (HT/T2DM). The anthropometric, clinical and biochemical characteristics of the study population according to glucose tolerance status are shown in Table 1. Of note, ferritin, transferrin, TIBC, and hs-CRP increased from the first to the third group (Table 1) whereas serum iron and hemoglobin showed an opposite pattern (Table 1). It’s important to remark that hemoglobin and ferritin levels of all study population were within the normal range. Figure 1 reports two histograms plotting the distributions of ferritin levels separately in males and females.

### Hemodynamic parameters

Peripheral and aortic hemodynamic parameters for the study population, according to glucose tolerance groups, are presented in Table 2. There were no significant differences among groups for peripheral BP values and heart rate. On the contrary, from the first to the third group of glucose tolerance, we noted a significant increase of central (c)-SBP and c-PP, without significant difference for c-DBP. Hypertensive T2DM patients showed a significantly higher c-SBP and c-PP values than HT/NGT, but similar to HT/IGT. Similarly, all indices of vascular stiffness progressively increased from the first to third group. In particular, PWV values were significantly higher in HT/IGT and HT/T2DM patients in comparison with HT/NGT, but without any significant differences between these two groups. Similarly, also AI and AP values were significantly higher in HT/IGT and HT/T2DM patients than HT/NGT but substantially comparable between them (Table 2).

**Table 2:** Peripheral and aortic hemodynamic parameters of the study population according to glucose tolerance status

	All (n = 462)	HT/NGT (n = 271)	HT/IGT (n = 146)	HT/T2DM (n = 45)	p
Heart rate, bpm	69.7 ± 12.9	69.2 ± 13.9	70.1 ± 10.2	71.1 ± 14.2	0.578
Systolic BP, mmHg	144.5 ± 22.1	144.3 ± 22.2	144.6 ± 21.4	145.7 ± 23.5	0.927
Diastolic BP, mmHg	90.3 ± 7.8	90.2 ± 8.3	90.4 ± 7.6	91.2 ± 5.5	0.724
PP, mmHg	54.2 ± 23.2	54.1 ± 23.5	54.1 ± 22.4	54.5 ± 24.8	0.994
c- systolic BP, mmHg	133.3 ± 11.3	132.2 ± 11.5	133.9 ± 10.6	138.4 ± 10.9	0.002
c- diastolic BP, mmHg	90.3 ± 8.1	90.4 ± 8.6	90.1 ± 7.3	90.8 ± 7.6	0.855
c-PP, mmHg	42.9 ± 13.5	41.7 ± 13.7	43.8 ± 12.8	47.5 ± 13.4	0.019
AP, mmHg	10.8 ± 6.9	9.6 ± 6.8	12.3 ± 6.5	13.4 ± 7.2	< 0.0001
AI, %	25.2 ± 13.3	22.8 ± 13.3	28.1 ± 12.6	30.3 ± 12.8	< 0.0001
PWV, m/s	7.5 ± 2.0	7.2 ± 1.5	7.7 ± 2.3	8.4 ± 2.9	< 0.0001

Data are mean and standard deviation

*H* hypertensive, *NGT* normal glucose tolerance, *T2DM* type 2 diabetes mellitus, *BP* blood pressure, *c* central, *PP* pulse pressure, *AP* augmentation pressure, *AI* augmentation index, *PWV* pulse wave velocity

### Correlation analysis

**Fig. 2:**
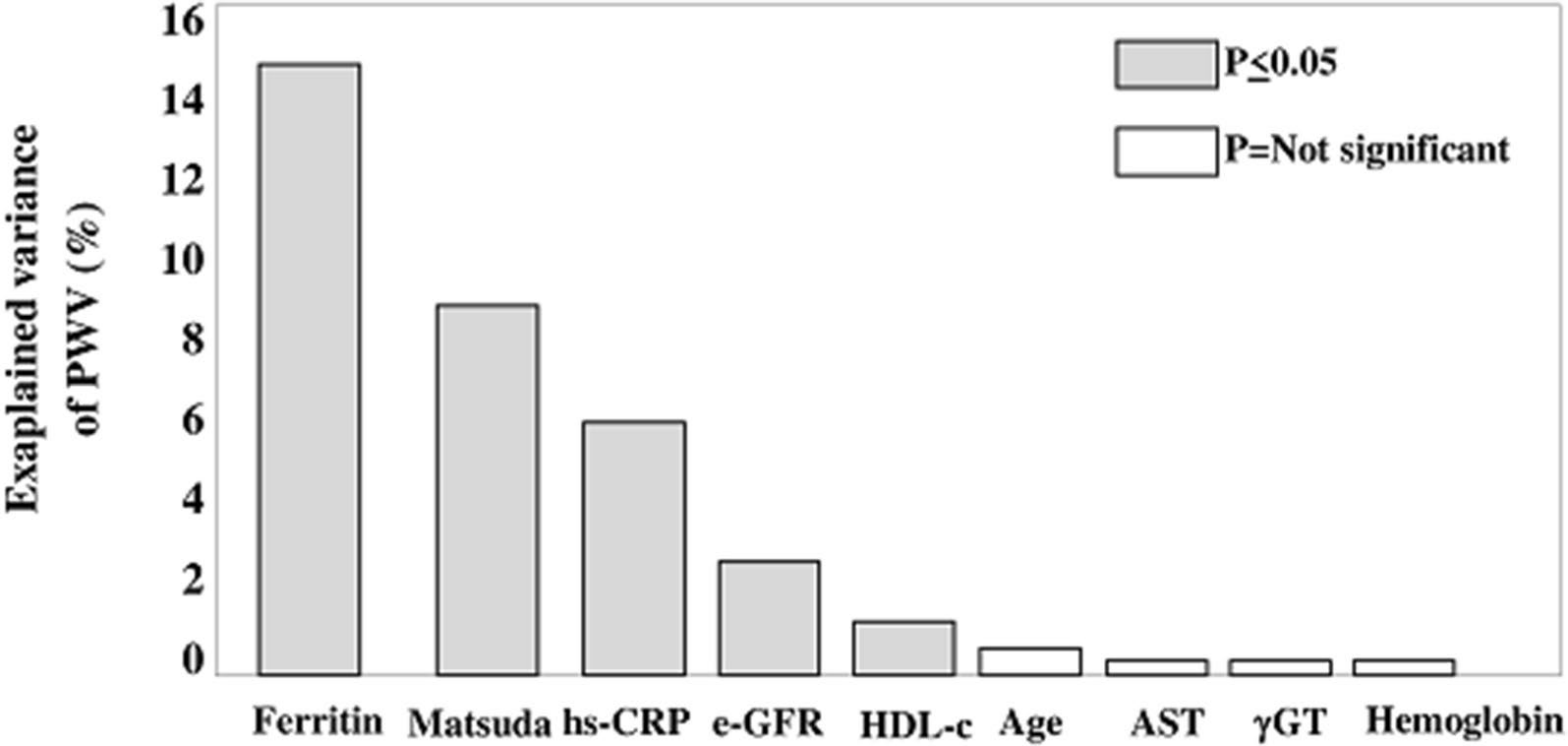
Percentage explained variance of pulse wave velocity (PWV) explained by each covariate into the multiple model (see Main effects model in Table 3). For example, the explained variance in PWV explained by ferritin is calculated by elevating at squared the partial correlation coefficient of the ferritin-PWV link (partial r = 0.385). The partial correlation coefficient explains the correlation between ferritin and PWV holding constant the other covariates for both variables. The squared partial correlation (0.385^2^ = 0.149, 14.9%) expresses how much of the variability in the PWV which is not explained by other covariates is explained by ferritin (see also “Methods”—“Statistical analysis”). *Hs-CRP* high sensitivity C reactive protein, *e-GFR* estimated glomerular filtration rate, *HDDL-c* high density lipoprotein cholesterol, *AST* aspartate aminotransferase, *γGT* γ-glutamyltransferase

A linear correlation analysis was performed to assess the association between PWV and all variables listed in Table 1. PWV was related directly with ferritin, hs-CRP, age, insulin, AST, γGT, transferrin, and hemoglobin and inversely with the MATSUDA index, serum iron, e-GFR, and HDL cholesterol. In multiple linear regression model (Table 3) testing a series of univariate correlates of PWV (according to the strategy described in “Methods” section—“Statistical analysis”), ferritin was the strongest correlate of PWV explaining a 14.9% of the variance of this biomarker, followed by MATSUDA index (9.0%), hs-CRP (5.6%), e-GFR (1.9%), and HDL cholesterol (0.9%) (Fig. 2). Age, AST, γGT, and hemoglobin did not significantly contribute to explain the variance of PWV (Fig. 2). Overall, the model was able to explain a 39.9% of PWV variability.

**Table 3:** Multiple linear regression analyses of PWV (see “Methods”—“Statistical analysis” for details)

Dependent variable: PWV	Main effects model	Model with effect modification
	Beta (*p* value)	Beta (*p* value)
Age, years	0.06 (0.13)	0.07(0.10)
MATSUDA	− 0.26 (< 0.001)	− 0.27 (< 0.001)
HDL cholesterol, mg/dl	0.08 (0.04)	0.07 (0.06))
Hemoglobin, g/dl	0.007 (0.85)	0.02 (0.60)
AST, U/l	0.04 (0.35)	0.03 (0.41)
γGT, U/l	− 0.02 (0.62)	− 0.03 (0.47)
e-GFR, ml/min/1.73 m^2^	− 0.11 (0.003)	− 0.10 (0.009)
Ferritin, ng/ml	0.37 (< 0.001)	0.19 (0.01)
hs-CRP, mg/l	0.20 (< 0.001)	0.05 (0.42)
Ferritin * hs-CRP	…	0.28 (0.004) (see Fig. 2)

Data are standardised regression coefficients (beta) and p values

By forcing gender into the main effect model does not modify the ferritin-PWV relationship (beta = 0.39, p < 0.001) and this was also true when forcing transferrin saturation (beta = 0.37, p < 0.001). In the same model, neither gender (beta = − 0.06, p = 0.16) nor transferrin saturation (beta = 0.07, p = 0.08) were related to PWV

*PWV* pulse wave velocity, *HDL* high density lipoproteins, *hs-CRP* high sensitivity C reactive protein, *AST* aspartate aminotransferase, *γGT* γ-glutamyltransferase, *e-GFR* estimated glomerular filtration rate

### Effect modification analysis

**Fig. 3:**
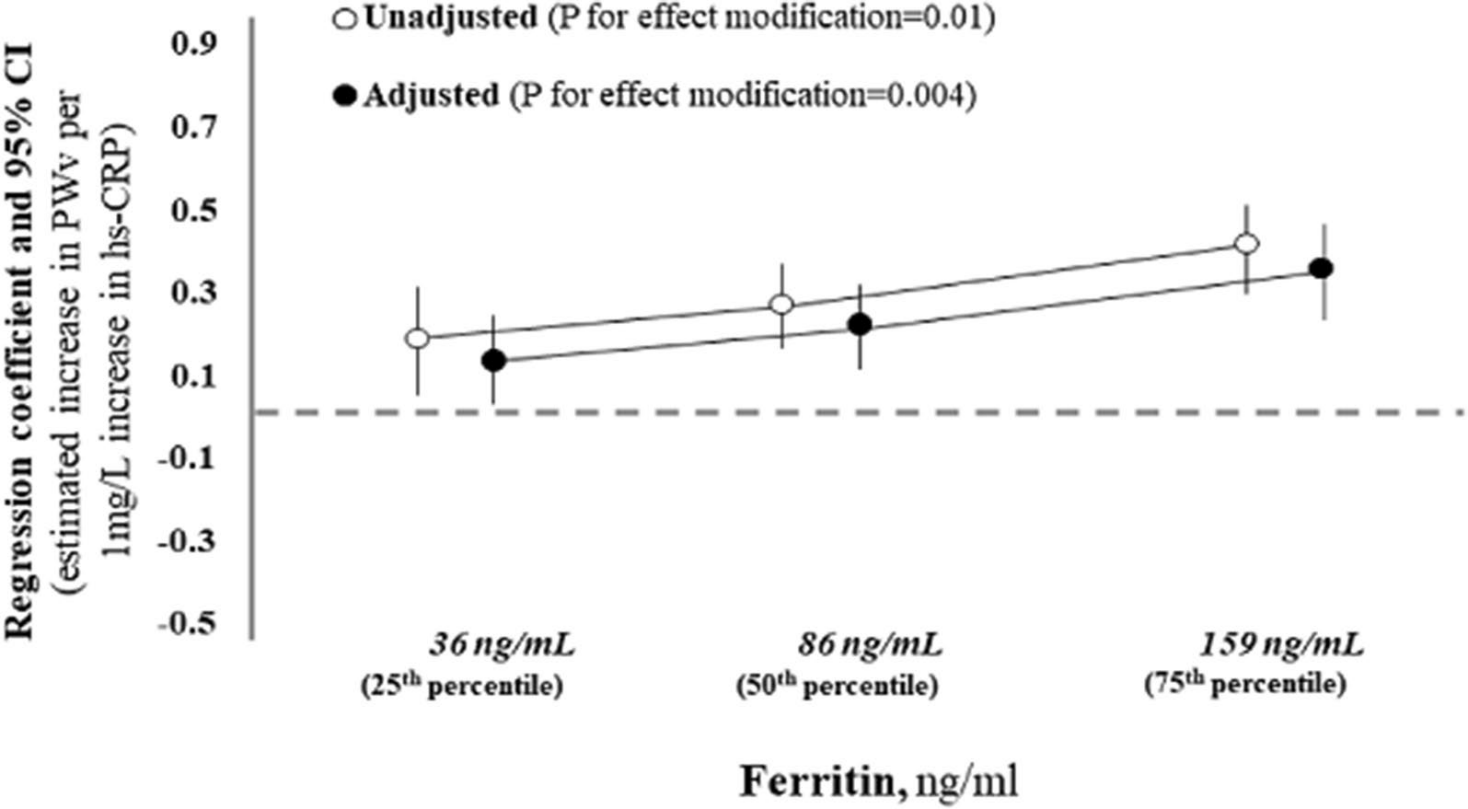
Effect modification by ferritin levels on the relationship between high sensitivity C reactive protein and pulse wave velocity (PWV). Given the fact that an effect modification by ferritin exists (see Table 3), the estimated increase in PWV associated to 1 mg/l increase in hs-CRP must be calculated at predefined values of the effect modifier. Thus, in crude terms, when ferritin is 36 ng/ml (the 25th percentile), 86 ng/ml (the 50th percentile) and 159 ng/ml (the 75th percentile), the estimated increase in PWV associated to 1 mg/l increase in hs-CRP is 0.18 m/s, 0.26 m/s and 0.37 m/s, respectively. These three effects associated to a fixed increase in CRP (1 mg/l) statistically differ with a p value of 0.01. Data are crude and adjusted (Table 3—Model with effect modification) estimates. See “Methods”—“Statistical analysis” for more details

Circulating levels of ferritin were directly related to those of hs-CRP (r = 0.20, p < 0.001). Thus, in Table 3 the effect modification by ferritin levels on the hs-CRP-PWV relationship was investigated by crude and adjusted analyses (see “Methods” section, “Statistical analysis” for details). On crude terms, serum ferritin was a strong modifier of the relationship between hs-CRP and PWV (P for effect modification = 0.01 and Fig. 3). The effect modification by ferritin on the hs-CRP-PWV relationship remained significant also after data adjustment for potential confounders (P for effect modification = 0.004, Table 3 and Fig. 3). As shown in Fig. 3, the estimated increase in PWV value portended by a fixed increase in hs-CRP (+ 1 mg/l) was progressively higher across increasing values of serum ferritin. These results indicate that high serum ferritin levels are not only a risk factor of arterial stiffness, but also amplify the harmful effect of inflammation on PWV.

## Discussion

In this study, in patients with a wide range of glucose tolerance (from NGT to newly diagnosed T2DM), we found that ferritin levels increase in parallel to the worsening of glycometabolic parameters (particularly insulin sensitivity), the raise of inflammation (as assessed by hs-CRP) and the reduction of serum iron and hemoglobin levels (Table 1). These observations go in close parallellism with the increase of central hemodynamic parameters and arterial stiffness indicators, in particular carotid-femoral PWV, across the same groups (from NGT to T2DM). We also found that, in a multiple linear regression model, serum ferritin resulted to be the strongest determinant of PWV explaining a 14.9% of the variability of this biomarker and the ferritin-PWV relationship remained significant also after data adjustment for well-known modulators of arterial stiffness such as insulin sensitivity, hs-CRP and renal function. Another novel hypothesis generated by us, is that serum ferritin could act as an effect modifier on the relationship between inflammation and arterial stiffness in the same patient-population. Thus, the present results suggest that serum ferritin levels are not only a risk factor of arterial stiffness but also an amplifier of the damage induced by inflammation on PWV.

### Ferritin, glucose metabolism and atherosclerosis: a complex matter

In recent years the interrelationship between ferritin, glucose metabolism, arterial stiffness and inflammation has been extensively investigated. Scientific evidence has shown that high glucose levels also in the prediabetic range and insulin resistance might lead to increased arterial stiffness and cardiac concentric remodeling [27], moreover in a sample of hypertensive patients a significant positive association between baseline brachial-ankle PWV and the risk of new-onset diabetes was found [28]. Also serum ferritin levels have been associated with new-onset T2DM risk in Chinese [29] and Korean [30] populations and with increased arterial stiffness in healthy Korean adults [20]. Moreover, besides classical confounders, 24-h ambulatory BP and hs-CRP levels were determinants of pharmacologically (empagliflozin) induced improvement of arterial stiffness parameters [31]. Furthermore, a strong association between serum ferritin levels and atherosclerotic injury has been reported in different settings of patients [32, 33]. In particular, increased common carotid artery intima-media thickness and presence of carotid plaques has been associated with serum ferritin in haemodialysis patients [32] and patients with non-alcoholic fatty liver disease [33]. However, there is growing evidence demonstrating a strong association between higher serum ferritin levels and functional vascular damage. According with this, Ha et al. reported, in a large cohort of healthy subjects, an association between ferritin and brachial-ankle PWV [20]. Moreover in haemodialysis patients, serum ferritin was associated with progressive arterial stiffness increase during a 3 years follow-up, but only for serum values > 500 ng/ml [21]. In hypertensive patients, only few data are reported in literature. In this regard, Valenti and co-authors [18] showed that hyperferritinemia (i.e. higher than 240 ng/ml in females and 320 ng/ml in males) was associated with high aortic stiffness measured as carotid-femoral PWV in patients with well-controlled hypertensives but with several comorbidities and already receiving drug treatment whereas data about the glycometabolic state were not available in this study. All these factors may explain why hs-CRP was not retained as an independent risk factor of PWV in the multiple regression analysis [18]. In respect to the study by Valenti et al. our study has the advantage to include younger naive hypertensive, without chronic conditions (in particular renal and liver diseases) and who received no pharmacological treatment thus avoiding/minimizing possible confounding factors. Remarkably, all patients were evaluated by OGTT to define glucose tolerance and the effect of insulin sensitivity on vascular damage was assessed by Matsuda index, a strong surrogate of euglycemic clamp. Finally, the most important observation was that, in our study, serum ferritin levels were within the normal range value.

### Ferritin and increased PWV: the role of subclinical inflammation

Patients included in our study present iron levels within the normal range and for this reason the association between ferritin and PWV cannot be explained by iron overload. However, it’s known that ferritin is strictly regulated by cytokines at various levels (transcriptional, posttranscriptional and translational) during cellular differentiation, proliferation and inflammatory burden [34]. In particular, it has been demonstrated that tumor necrosis factor alpha (TNFα) and interleukin 1a (IL1α) are able to induce the expression of the H chain of ferritin at the transcriptional level in different cell types [35]. Moreover, cytokines have also posttranscriptional effects on ferritin expression. According with this, translation of ferritin is induced by IL-1α, IL-6 or TNFα in the HepG2 hepatic cell line [35, 36], and iron is required for this action in fact the ferritin upregulation may be inhibited by deferoxamine (an intracellular iron chelator) [35]. These studies along with cytokine-mediated regulation suggest that inflammation can affect ferritin regulation. Thus, it’s plausible to speculate that in chronic conditions characterized by subclinical inflammation, such as arterial hypertension and T2DM, dysregulation of iron metabolism may lead to increased oxidative stress promoting arterial stiffening. Our results and interpretation are in keeping with those reported in two previous population-based studies [37, 38]. In a paper by Wolff et al. [37], in both men and women a dose–response relationship was found between serum ferritin levels and carotid atherosclerosis, so that higher serum ferritin levels were associated with greater risk of carotid plaque development. In another paper by Ahluwalia et al. [38], a significant interaction was found between inflammation and ferritin levels to explain the severity of carotid atherosclerosis in the study sample. Given the fact that the effect modification is a mutualistic phenomenon, Ahluwalia et al. proposed the hypothesis that inflammation may act as an effect modifier of the relationship between ferritin and PWV [38].

### Ferritin as a trigger of inflammation

It is well known that serum ferritin is an acute-phase reactant, reflecting the degree of acute and chronic inflammation in several systemic diseases. However, emerging evidence suggests a potential causative role for ferritin in the inflammatory process [4]. In this regard, in vitro studies showed that ferritin may work as a local cytokine, activating Mitogen-Activated Protein Kinases (MAPK)-induced NF-kB in an iron-independent manner. This activity induces the increased expression of multiple pro-inflammatory mediators and a near 100-fold increase in inducible NO synthase which amplify oxidative stress [39, 40]. In addition, ferritin is also able to directly modulate the lymphocyte function thus acting as pathogenetic player in the innate immune response [41]. Considering that our study population presented a normal inflammation and iron status, it is also clinically plausible that ferritin per se might affect the increase in oxidative stress promoting those mechanisms that lead to vascular remodeling and increased arterial stiffness. Clinical practice supports this hypothesis, because ferritin represents a biomarker of disease progress and an independent predictor of various clinical outcome in different settings of patients [42–44]. Since the increase in carotid femoral PWV is associated with a significant risk increase for CV morbidity and mortality, we can speculate that the association between serum ferritin levels and PWV may be responsible for the negative prognostic effect of ferritin. Overall, the studies performed so far do not fully clarify which is the cause and which is the effect in the complex pathway involving ferritin, inflammation and atherosclerosis. For this reason, any hypothesis is purely speculative and the exact mechanisms underlying the relationship between ferritin and carotid atherosclerosis in humans with normal iron levels still remains to be fully elucitaded.

## Conclusion

In conclusion, serum ferritin levels, within the normal range, represent an independent risk factor of arterial stiffness in a large cohort of naïve hypertensive patients with different glucose tolerance and a strong effect modifier on the relationship between inflammation and PWV, thus a possible candidate for early marker of atherosclerosis at least in this setting of patients. This may be clinically relevant because ferritin is an easily determined laboratory parameters and widely used in medical practice.

The main limitation of the study is its cross-sectional design, therefore no causal link can be claimed. In addition the measurements of hepcidin, a well-known regulator of body iron fluxes, were not available. This may be important because the determination of hepcidin levels could give more information about iron metabolism, also in subclinical inflammation. However, we evaluated transferrin saturation which is an important determinant of hepcidin release.

## Data Availability

The datasets used and analysed during the current study are available from the corresponding author on reasonable request.

## Abbreviations

ROS: Reactive oxygen species
NO: Nitric oxide
CV: Cardiovascular
H: Hypertensive
NGT: Normal glucose tolerance
T2DM: Type 2 diabetes mellitus
OGTT: Oral glucose tolerance test
SBP: Systolic blood pressure
DBP: Diastolic blood pressure
PWV: Pulse wave velocity
BMI: Body mass index
LDL: Low density lipoproteins
HDL: High density lipoproteins
hs-CRP: High sensitivity C reactive protein
TIBC: Transferrin iron binding capacity
AST: Aspartate aminotransferase
ALT: Alanine aminotransferase
γ-GT: γ-glutamyltransferase
e-GFR: Estimated glomerular filtration rate
BP: Blood pressure
c: Central
PP: Pulse pressure
AP: Augmentation pressure
AI: Augmentation index

## References

[1] Marques VB, Nascimento TB, Ribeiro RF, Broseghini-Filho GB, Rossi EM, Graceli JB (2015). Chronic iron overload in rats increases vascular reactivity by increasing oxidative stress and reducing nitric oxide bioavailability. Life Sci.

[2] Oboshi M, Naito Y, Sawada H, Iwasaku T, Okuhara Y, Eguchi A (2016). Attenuation of hypertension and renal damage in renovascular hypertensive rats by iron restriction. Hypertens Res.

[3] Shim YS, Kang MJ, Oh YJ, Baek JW, Yang S, Hwang IT (2017). Association of serum ferritin with insulin resistance, abdominal obesity, and metabolic syndrome in Korean adolescent and adults the Korean National Health and Nutrition Examination Survey, 2008 to 2011. Med (United States).

[4] Kernan KF, Carcillo JA (2017). Hyperferritinemia and inflammation. Int Immunol.

[5] Ebrahimi KH, Hagedoorn PL, Hagen WR (2015). Unity in the biochemistry of the iron-storage proteins ferritin and bacterioferritin. Chem Rev.

[6] Arosio P, Elia L, Poli M (2017). Ferritin, cellular iron storage and regulation. IUBMB Life.

[7] Cho MR, Park JK, Choi WJ, Cho AR, Lee YJ (2017). Serum ferritin level is positively associated with insulin resistance and metabolic syndrome in postmenopausal women: a nationwide population-based study. Maturitas..

[8] Chen L, Li Y, Zhang F, Zhang S, Zhou X, Ji L (2018). Elevated serum ferritin concentration is associated with incident type 2 diabetes mellitus in a Chinese population: a prospective cohort study. Diabetes Res Clin Pract.

[9] Wang YL, Koh WP, Yuan JM, Pan A (2017). Plasma ferritin, C-reactive protein, and risk of incident type 2 diabetes in Singapore Chinese men and women. Diabetes Res Clin Pract.

[10] Ryoo JH, Kim SY, Oh CM, Park SK, Kim E, Park SJ (2015). The incidental relationship between serum ferritin levels and hypertension. Int J Cardiol.

[11] Kim MK, Baek KH, Song KH, Kang MIL, Choi JH, Bae JC (2012). Increased serum ferritin predicts the development of hypertension among middle-aged men. Am J Hypertens..

[12] Ettehad D, Emdin CA, Kiran A, Anderson SG, Callender T, Emberson J (2016). Blood pressure lowering for prevention of cardiovascular disease and death: a systematic review and meta-analysis. Lancet.

[13] Sciacqua A, Maio R, Miceli S, Pascale A, Carullo G, Grillo N (2012). Association between one-hour post-load plasma glucose levels and vascular stiffness in essential hypertension. PLoS ONE.

[14] Avolio AP, Van Bortel LM, Boutouyrie P, Cockcroft JR, McEniery CM, Protogerou AD (2009). Role of pulse pressure amplification in arterial hypertension: experts’ opinion and review of the data. Hypertension.

[15] Ben-Shlomo Y, Spears M, Boustred C, May M, Anderson SG, Benjamin EJ (2014). Aortic pulse wave velocity improves cardiovascular event prediction: an individual participant meta-analysis of prospective observational data from 17,635 subjects. J Am Coll Cardiol.

[16] Mattace-Raso FUS, Van Der Cammen TJM, Hofman A, Van Popele NM, Bos ML, Schalekamp MADH (2006). Arterial stiffness and risk of coronary heart disease and stroke: the Rotterdam Study. Circulation.

[17] Vlachopoulos C, Aznaouridis K, Stefanadis C (2010). Prediction of cardiovascular events and all-cause mortality with arterial stiffness. A systematic review and meta-analysis. J Am Coll Cardiol..

[18] Valenti L, Maloberti A, Signorini S, Milano M, Cesana F, Cappellini F (2015). Iron stores, hepcidin, and aortic stiffness in individuals with hypertension. PLoS ONE..

[19] Prats-Puig A, Moreno M, Carreras-Badosa G, Bassols J, Ricart W, López-Bermejo A (2016). Serum ferritin relates to carotid intima-media thickness in offspring of fathers with higher serum ferritin levels. Arterioscler Thromb Vasc Biol.

[20] Ha JY, Kim MK, Kang S, Nam JS, Ahn CW, Kim KR (2016). Serum ferritin levels are associated with arterial stiffness in healthy Korean adults. Vasc Med (United Kingdom)..

[21] Lin KC, Tsai MY, Chi CL, Yu LK, Huang LH, Chen CA (2015). Serum ferritin is associated with arterial stiffness in hemodialysis patients: results of a 3-year follow-up study. Int Urol Nephrol.

[22] Williams B, Mancia G, Spiering W, Rosei EA, Azizi M, Burnier M (2018). ESC/ESHGuidelines for themanagement of arterial hypertension. J Hypertens.

[23] Matsuda M, DeFronzo RA (1999). Insulin sensitivity indices obtained from oral glucose tolerance testing: comparison with the euglycemic insulin clamp. Diabetes Care.

[24] Levey AS, Stevens LA, Schmid CH, Zhang Y, Castro AF, Feldman HI (2009). A new equation to estimate glomerular filtration rate. Ann Intern Med.

[25] Chen CH, Ting CT, Nussbacher A, Nevo E, Kass DA, Pak P (1996). Validation of carotid artery tonometry as a means of estimating augmentation index of ascending aortic pressure. Hypertension.

[26] De Mutsert R, Jager KJ, Zoccali C, Dekker FW (2009). The effect of joint exposures: examining the presence of interaction. Kidney Int.

[27] Markus MRP, Rospleszcz S, Ittermann T, Baumeister SE, Schipf S, Siewert-Markus U (2019). Glucose and insulin levels are associated with arterial stiffness and concentric remodeling of the heart. Cardiovasc Diabetol.

[28] Zhang Y, He P, Li Y, Zhang Y, Li J, Liang M (2019). Positive association between baseline brachial–ankle pulse wave velocity and the risk of new-onset diabetes in hypertensive patients. Cardiovasc Diabetol.

[29] Gao S, Zhao D, Qi Y, Wang M, Zhao F, Sun J (2017). The association between serum ferritin levels and the risk of new-onset type 2 diabetes mellitus: a 10-year follow-up of the chinese multi-provincial cohort study. Diabetes Res Clin Pract.

[30] Kim S, Park SK, Ryoo JH, Choi M, Hong HP, Park JH (2015). Incidental risk for diabetes according to serum ferritin concentration in Korean men. Clin Chim Acta.

[31] Bosch A, Ott C, Jung S, Striepe K, Karg MV, Kannenkeril D (2019). How does empagliflozin improve arterial stiffness in patients with type 2 diabetes mellitus? Sub analysis of a clinical trial. Cardiovasc Diabetol..

[32] Drüeke T, Witko-Sarsat V, Massy Z, Descamps-Latscha B, Guerin AP, Marchais SJ (2002). Iron therapy, advanced oxidation protein products, and carotid artery intima-media thickness in end-stage renal disease. Circulation.

[33] Valenti L, Swinkels DW, Burdick L, Dongiovanni P, Tjalsma H, Motta BM (2011). Serum ferritin levels are associated with vascular damage in patients with nonalcoholic fatty liver disease. Nutr Metab Cardiovasc Dis..

[34] You SA, Wang Q (2005). Ferritin in atherosclerosis. Clin Chim Acta.

[35] Muntane-Relat J, Ourlin JC, Domergue J, Maurel P (1995). Differential effects of cytokines on the inducible expression of CYP1A1, CYP1A2, and CYP3A4 in human hepatocytes in primary culture. Hepatology.

[36] Tran TN, Eubanks SK, Schaffer KJ, Zhou CY, Linder MC (1997). Secretion of ferritin by rat hepatoma cells and its regulation by inflammatory cytokines and iron. Blood.

[37] Wolff B, Völzke H, Lüdemann J, Robinson D, Vogelgesang D, Staudt A (2004). Association between high serum ferritin levels and carotid atherosclerosis in the study of health in pomerania (SHIP). Stroke.

[38] Ahluwalia N, Genoux A, Ferrieres J, Perret B, Carayol M, Drouet L (2010). Iron status is associated with carotid atherosclerotic plaques in middle-aged adults. J Nutr.

[39] Ruddell RG, Hoang-Le D, Barwood JM, Rutherford PS, Piva TJ, Watters DJ (2009). Ferritin functions as a proinflammatory cytokine via iron-independent protein kinase C zeta/nuclear factor kappaB—regulated signaling in rat hepatic stellate cells. Hepatology.

[40] Freeman GJ, Casasnovas JM, Umetsu DT, Dekruyff RH (2010). TIM genes: a family of cell surface phosphatidylserine receptors that regulate innate and adaptive immunity. Immunol Rev.

[41] Gray CP, Arosio P, Mersey P (2002). Heavy chain ferritin activates regulatory T cells by induction of changes in dendritic cells. Blood.

[42] Horvat CM, Bell J, Kantawala S, Au AK, Clark RSB, Carcillo JA (2019). C-reactive protein and ferritin are associated with organ dysfunction and mortality in hospitalized children. Clin Pediatr (Phila)..

[43] Kadoglou NPE, Biddulph JP, Rafnsson SB, Trivella M, Nihoyannopoulos P, Demakakos P (2017). The association of ferritin with cardiovascular and all-cause mortality in community-dwellers: the English longitudinal study of ageing. PLoS ONE..

[44] Ma L, Zhao S (2017). Risk factors for mortality in patients undergoing hemodialysis: a systematic review and meta-analysis. Int J Cardiol.

